# Omics Meets Biology: Application to the Design and Preclinical Assessment of Antivenoms

**DOI:** 10.3390/toxins6123388

**Published:** 2014-12-15

**Authors:** Juan J. Calvete, Libia Sanz, Davinia Pla, Bruno Lomonte, José María Gutiérrez

**Affiliations:** 1 Structural and Functional Venomics Lab, Instituto de Biomedicina de Valencia (CSIC), Valencia 46010, Spain; E-Mails: libia.sanz@ibv.csic.es (L.S.); dpla@ibv.csic.es (D.P.); 2 Instituto Clodomiro Picado, Facultad de Microbiología, Universidad de Costa Rica, San José 11501, Costa Rica

**Keywords:** snake venom, snakebite envenoming, antivenom, preclinical venom neutralization assays, venomics, antivenomics

## Abstract

Snakebite envenoming represents a neglected tropical disease that has a heavy public health impact worldwide, mostly affecting poor people involved in agricultural activities in Africa, Asia, Latin America and Oceania. A key issue that complicates the treatment of snakebite envenomings is the poor availability of the only validated treatment for this disease, antivenoms. Antivenoms can be an efficacious treatment for snakebite envenoming, provided they are safe, effective, affordable, accessible and administered appropriately. The shortage of antivenoms in various regions, particularly in Sub-Saharan Africa and some parts of Asia, can be significantly alleviated by optimizing the use of current antivenoms and by the generation of novel polyspecific antivenoms having a wide spectrum of efficacy. Complementing preclinical testing of antivenom efficacy using *in vivo* and *in vitro* functional neutralization assays, developments in venomics and antivenomics are likely to revolutionize the design and preclinical assessment of antivenoms by being able to test new antivenom preparations and to predict their paraspecific neutralization to the level of species-specific toxins.

## 1. Introduction: Snakebite Envenoming and the Challenge of Generating Effective Antivenoms

Snakebite envenoming is largely a neglected threat to public health that affects some of the world’s poorest rural communities, mainly those involved in subsistence farming and livestock grazing activities in tropical and subtropical regions of Africa, Asia, Latin America and Oceania. An estimated 5.5 million people are bitten by snakes each year, resulting in about 400,000 amputations and over 100,000 deaths; however, the true scale of this “disease of poverty” may be much greater than these hospital-based statistics, since many affected people do not attend health facilities [1,2,3,4]. Persistent underestimation of their true morbidity and mortality has made snakebites the most neglected of all of the World Health Organization (WHO)’s “neglected tropical diseases”, downgrading its public health importance. Moreover, the WHO recently moved snakebite envenoming to the category of “neglected tropical condition”, further reducing its relevance and thereby affecting the global awareness of the magnitude of this pathology. Strategies to address this neglect should include the improvement of affordable, effective antivenoms [3,5,6,7,8], the timely administration of which remains the only specific treatment for envenoming [9], more than a century after the development of the first serum antivenimeux by Calmette [10,11,12] and Phisalix and Bertrand [13,14] simultaneously, but independently, in 1894. In addition, the struggle against snakebite envenoming should include other aspects, such as improved statistics on morbidity and mortality, adequate access and distribution of safe and effective antivenoms in the public health system, training of health staff in the effective management of this disease and promotion of preventive campaigns at the local community level, among other goals. This series of tasks demand, in turn, the involvement of a large spectrum of stakeholders in diverse fields.

More than 45 commercial or government antivenom producers exist around the world [15]. However, the lack of financial incentives in a technology that has remained relatively unchanged for the better part of the second half of the 20th Century, along with dwindling markets and a lack of leadership from global public health organizations, have made antivenom production a field of limited improvements and very little innovation [7]. Snake antivenoms became scarce or non-existent as poor commercial incentives forced some manufacturers to leave the market and others to downscale production or increase the price, leading to a decline in the availability and accessibility for these life-saving antidotes to the millions of rural poor most at risk from snakebites in low and middle-income countries [3]. Furthermore, many manufacturing laboratories in public institutions have suffered a lack of investment and renewal of their technological platforms, together with deficient training of their staff, thereby affecting the quality and quantity of their antivenoms. To raise the awareness of public health authorities on the relevance of the snakebite problem and to ensure supplies of effective antivenoms in deficitary parts of the world, several initiatives have emerged in the last decade. In 2008, members of the international toxinology community established the Global Snakebite Initiative [16,17]. Some of the goals of this organization include advocacy on the seriousness of this health problem, together with the promotion of research initiatives to improve epidemiological and clinical knowledge of envenomings and initiatives to improve training of health staff and antivenom quality control and accessibility in some regions of the world [2,5,18,19]. The GSI strategy has also the goal of coupling modern proteomics, immunological, pharmacological and molecular biological techniques to the quest for improved therapeutics and the understanding of the underlying pathophysiology and clinical manifestations of snakebite envenomings [4,20,21,22,23].

**Figure 1:**
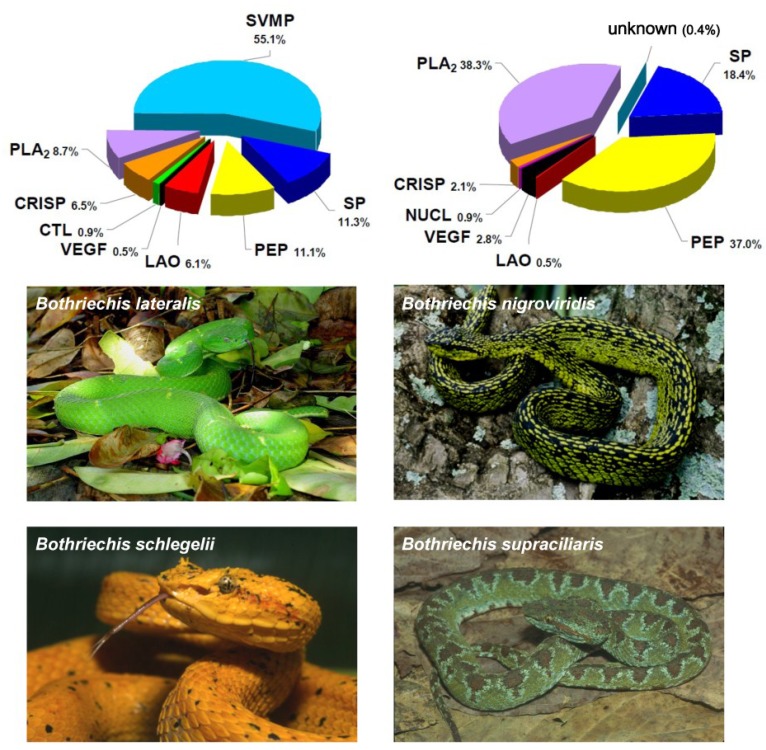

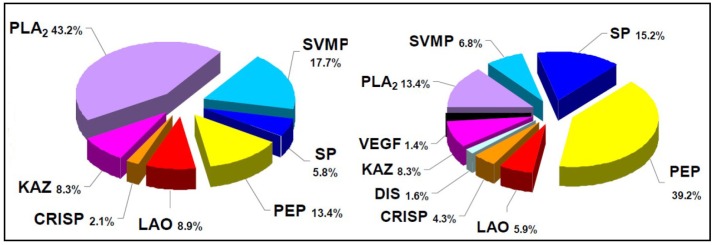
Highly divergent toxin compositions in phylogenetically-close snake taxa. Venom components of four *Bothriechis* species that inhabit Costa Rica were assigned to protein families, and their abundances were estimated, by using the “snake venomics” analytical strategy. As shown in the corresponding pie charts summarizing protein family abundances (%), the venom of *Bothriechis lateralis* is dominated by metalloproteinases, whereas small peptides of the vasoactive type are predominant in the venom of *B. supraciliaris*. On the other hand, *B. schlegelii* venom contains the highest proportion of phospholipases A_2_, while *B. nigroviridis* venom completely lacks metalloproteinases and presents a high percentage of an unusual phospholipase A_2_ recently characterized as a crotoxin-like complex, found for the first time in a non-rattlesnake New World pit viper. In addition, a novel protein type for snake venoms (Kazal-type proteinase inhibitor-like) was found in *B. schlegelii* and *B. supraciliaris*, but not in the other two species of this genus. Protein family abbreviations correspond to: SVMP, metalloproteinase; PLA_2_, phospholipase A_2_; SP, serine proteinase; CRISP, cysteine-rich secretory proteins; CTL, C-type lectins/lectin-like; VEGF, vascular endothelial growth factor; LAO, L-amino acid oxidase; NUCL, nucleotidase; DIS, disintegrin; PEP, small peptide; KAZ, Kazal-type proteinase inhibitor-like. Data adapted from Lomonte *et al*. [32].

A key technical issue concerning the generation of new antidotes for snakebite envenoming is the design of improved immunization mixtures in such a way that the resulting antivenoms are effective against most venoms of the medically-relevant snake species within the geographical range where these antivenoms are intended to be used. This purpose is not trivial given the well-documented occurrence of venom variability at the genus, species, subspecies, population and individual levels [24]. The variability of venom composition may endow snakes with the capability to adapt to different ecological niches. This is clearly evident for highly adaptable snake species of a wide geographical distribution in which allopatric venom variation may result in variable clinical presentations of envenomings [23,25]. In addition, variation in venom composition is dictated by different genomic and postgenomic mechanisms [26,27], and phenotypic venom variation across conspecific populations often involves ontogenetic shifts in venom protein expression [23]. Furthermore, venom is evolutionarily a highly labile trait, even among very closely-related taxa [28,29], preventing the prediction of venom composition and toxic activity based on phylogenetic distance. For instance, species within the genus *Crotalus* express either Type I (high levels of metalloprotease and low toxicity) or Type II (low metalloprotease, high toxicity) venoms, which result in completely different envenomings from a pathophysiological standpoint, and these venom phenotypes exhibit no phylogenetic relationship [30]. Furthermore, the finding of different evolutionary solutions within arboreal *Bothriechis* taxa for the same trophic purpose [31] (Figure 1) strengthens the view that phylogeny cannot be invoked as the sole criterion for species selection for antivenom production.

The occurrence of ontogenetic, geographic and individual intraspecific venom variability highlights the necessity of using pooled venoms as a representative sample for antivenom manufacture, and a thorough study of clinical, epidemiological, immunological, proteomic and toxicological information may contribute to the design of the venom mixtures for immunization. These methodological approaches include classical biochemical and state-of-the-art proteomic analysis of venoms, the study of the toxicological profile of venom effects using *in vivo* and *in vitro* tests, and the investigation of the immunological cross-reactivity of antivenoms against homologous and heterologous venoms. Knowledge on the paraspecificity of antivenoms is not only of applied importance to optimize the production strategy of a novel antivenom, but also for predicting the full clinical range of existing antivenoms against homologous and heterologous venoms. To this end, a platform has been developed to explore the neutralizing ability and immunological cross-reactivity of antivenoms through a combination of methodologies that will be briefly discussed.

## 2. Biochemical and Toxinological Toolbox for the Preclinical Assessment of Antivenom Efficacy

The analysis of the ability of an antivenom to neutralize the most relevant toxic activities of the snake venoms for which it was designed is a preclinical requisite before it can go into clinical trials and is approved for medical use. Simple experimental protocols have been developed to assess the ability of antivenoms to neutralize the most relevant toxic effects of snake venoms [22,33,34,35,36,37]. The most widely-used protocol is based on the incubation of a fixed dose of venom and variable dilutions of antivenom, followed by the injection of aliquots of the mixtures in the corresponding assay systems [22,33]. Another experimental platform, which is not regularly used, but which is relevant when testing antivenoms of variable pharmacokinetic profiles, is based on the injection of venom, followed by the administration of antivenom by the intravenous route. This approach does not involve the mixture of venom and antivenom before injection and, consequently, reproduces more closely the actual dynamics of therapy in the clinical setting. 

Lethality is the single most important effect to be tested when analyzing venom toxicity and its neutralization by antivenoms. For the lethality neutralization assay, a “challenge dose”, which usually corresponds to 3 to 6 LD_50_s, depending on the laboratory, is mixed with various dilutions of the antivenom, and the mixtures are incubated (generally for 30 min at 37 °C). Control samples include venom incubated with saline solution instead of antivenom. The mixtures are then injected in mice, either by the intravenous or the intraperitoneal routes, and deaths occurring during a predefined time span (24 h or 48 h) are recorded. Neutralization is expressed as the median effective dose (ED_50_), *i.e.*, the antivenom/venom ratio in which 50% of the injected mice survive. Depending on the laboratory, ED_50_ is expressed in various ways, *i.e*., milligrams of venom neutralized per milligrams of antivenom proteins, milligrams of venom neutralized by milliliters of antivenom, milliliters of antivenom required to neutralize one milligram of venom or the number of LD_50_s of venom neutralized per milliliter antivenom. The assessment of the ability of antivenoms to neutralize lethality is routinely performed in the manufacturers’ quality control laboratories and by national regulatory agencies, as part of regular analyses of the antivenoms being manufactured, purchased and distributed. Unfortunately, some countries rely mostly on data reported by the manufacturers, rather than on control exerted by regulatory agencies. It is necessary to strengthen, through workshops and other activities, the national capacities to perform quality control of antivenoms worldwide.

The neutralization of venom lethality is, and will remain, the gold standard in the preclinical testing of antivenom efficacy. However, the study of the biochemical and toxicological complexity of snake venoms has shown clearly that, besides lethality, the venoms of many species induce additional toxic activities that play key roles in the pathophysiology of human envenoming. For example, envenomings by viperid snakes in many regions around the world are characterized by complex local tissue damage (myonecrosis, dermonecrosis, edema, hemorrhage, blistering) and by systemic disturbances (hemorrhage, coagulopathy, cardiovascular shock, acute kidney injury). Therefore, a more detailed analysis of the preclinical efficacy of antivenoms should encompass, in addition to lethality, the neutralization of these other clinically-relevant effects [33,34,35]. A series of simple *in vivo* and *in vitro* laboratory assays has been developed for the quantitative assessment of hemorrhagic, myotoxic, dermonecrotic, coagulant and defibrinogenating activities, among others [36,38,39]. Thus, although the routine quality control of antivenoms involves the neutralization of lethal activity, when a new antivenom is being developed or when an existing antivenom is introduced to a new geographical setting, a comprehensive preclinical analysis of neutralizing efficacy should be performed against the most relevant toxic effects of the most important snake venoms in that particular region.

The single most important effect in elapid snake venoms is neurotoxicity. Presynaptically-acting PLA_2_s disrupt the integrity of nerve terminal plasma membrane, and post-synaptically-acting 3FTxs bind with strong affinity to the nicotinic cholinergic receptor at the motor end-plate, hence causing flaccid paralysis, which may result in paralysis of respiratory muscles and death [40]. The preclinical efficacy of antivenoms against these venoms can be assessed by the neutralization of lethal effect (the ED_50_ test), since the end result of neurotoxicity is death. Alternatively, neurotoxic activity can be assessed by using *ex vivo* nerve-muscle preparations, such as the phrenic-nerve and biventer-cervicis preparations. Venoms of a number of spitting cobras in Africa and Asia induce predominantly a local necrotizing effect in humans [40,41]. In these cases, antivenoms should be assessed for their capacity to neutralize lethality and dermonecrosis [42]. Sea snake venoms may induce, in addition to a neurotoxic effect, a systemic myotoxic action, which might result in rhabdomyolysis. Hence, antivenoms should be tested for the neutralization of lethal and myotoxic activities [22]. Venoms of some land Australian elapids induce neurotoxicity, myotoxicity and coagulopathy; consequently, preclinical evaluation of antivenoms should include the neutralization of these effects [43]. On the other hand, the preclinical assessment of antivenoms against viperid venoms should include the analysis of the lethal, hemorrhagic, myotoxic, coagulant and defibrinogenating activities of venoms [20]. A brief description of some of these methodologies follows.

(1) Hemorrhagic activity: The most widely-used method is based on the intradermal injection of venom solutions, followed, several hours later, by the measurement of the area of the hemorrhagic spot in the inner side of the skin [36,44,45]. Venom activity is expressed as the minimum hemorrhagic dose (MHD), which corresponds to the dose of venom that induces a hemorrhagic halo of 10 mm in diameter. More recently, the analysis of systemic, *i.e.*, pulmonary, hemorrhage has been introduced. Mice are injected i.v. with venom, and one hour later, the animals are sacrificed and the thoracic cavity exposed for observation of hemorrhagic spots on the surface of the lungs. The minimum pulmonary hemorrhagic dose (MPHD) corresponds to the lowest amount of venom that induces hemorrhagic spots in the lungs of all mice injected [46].
(2) Myotoxic activity: Venom-induced skeletal muscle necrosis can be assessed by histological examination of muscle tissue injected with venom. Mice receiving an intramuscular injection of venom solution, for example in the gastrocnemius muscle, are sacrificed 24 h later, and the injected muscle is processed for histological analysis. The number of necrotic cells and the total number of muscle cells are quantified by microscopic assessment, and the myotoxic effect is expressed as the necrotic index, *i.e.*, the ratio of necrotic muscle fibers to total muscle fibers [47]. Venom activity can be expressed as the dose inducing a necrotic index of 0.5. Since histological analysis is time consuming and not all laboratories have facilities for tissue processing for histology, a convenient alternative for the histological analysis is the quantification of the activity of the plasmatic enzyme, creatine kinase (CK), which is released from muscle fibers when the plasma membrane of muscle cells is disrupted. Mice are injected intramuscularly, as described, and a blood sample is collected usually 3 h after injection. After separation of plasma by centrifugation, the plasma CK activity is quantified by using commercial kits. The minimum myotoxic dose (MMD) is defined as the dose of venom that increases the plasma CK activity four times as compared to mice injected with saline solution [48].
(3) Dermonecrotic activity: This effect is assessed in either rats or mice by carrying out intradermal injections of venom solutions, followed by the measurement of the necrotic area in the inner side of the skin 72 h after venom injection [36].
(4) Coagulant activity: *In vitro* coagulant activity of venoms is assessed by the addition of various doses of venom to samples of citrated human plasma, obtained from healthy donors, followed by the determination of clotting time. Activity is expressed as the minimum coagulant dose (MCD), defined as the dose of venom that induces clotting in 60 seconds [36,39]. For assessing the thrombin-like activity of venoms, a similar test is performed on fibrinogen solutions instead of plasma [36].
(5) Defibrinogenating activity: This is assessed in rats or mice by intravenous injection of venom solutions. After a defined period of time, a blood sample is collected, placed in a glass tube and incubated at room temperature for observation of clot formation. Activity is expressed as the minimum defibrinogenating dose (MDD), defined as the dose of venom that induces incoagulability, *i.e.*, blood remains unclottable, in all animals injected [36,39].
(6) Other tests: Assays for the determination of other toxic activities, such as edema-forming activity, *ex vivo* neurotoxic activity and thrombocytopenic effect, have been described [49,50,51]. The analysis of the neutralization of venom enzyme activities, including neutralization of proteinase, phospholipase A_2_ and hyaluronidase activities, has been also investigated [41,52,53]. Similarly to the neutralization of lethality, for the analysis of the neutralization of venom enzyme activities, various mixtures of venom:antivenom incubated for 30 min at 37 °C are tested, and neutralization of venom activity is expressed as ED_50_, *i.e.*, the antivenom/venom ratio in which the effect of venom is neutralized by 50% [54]. In the case of coagulant and defibrinogenating activities, neutralization is expressed as the effective dose (ED), defined as the antivenom/venom ratio at which the clotting time of plasma is prolonged three times when compared to plasma incubated with venom alone (for coagulant activity) or as the antivenom/venom ratio in which blood clots form in all animals injected (for defibrinogenating effect) [39].

## 3. Omics Toolbox for the Preclinical Assessment of Antivenom Efficacy: The Venomics-Antivenomics Platform

**Figure 2:**
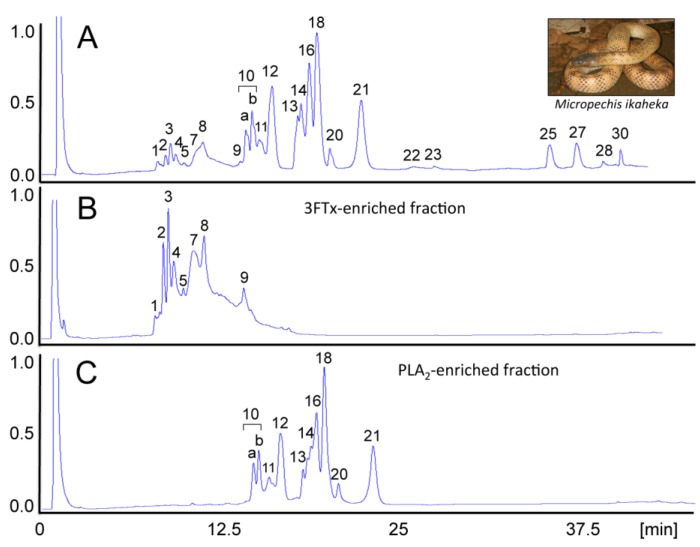
Venomics complements neutralization assays. Major effects of envenomings by the New Guinea small-eyed snake, *Micropechis ikaheka*, a large and powerfully-built elapid endemic to Papua New Guinea and Indonesian West Papua province, include life-threatening post-synaptic neuromuscular blockade, resulting in respiratory paralysis, PLA_2_-mediated myotoxicity, hypotension and cardiovascular abnormalities. The venom proteome of *M. ikaheka* is dominated by at least 29 D49-phospholipases A_2_ (PLA_2_) and 14 short and long neurotoxins of the three-finger toxin (3FTx) family [62]. These protein classes represent, respectively, 80% and 9.2% of the total venom proteins. Reverse-phase HPLC allowed the fractionation of *M. ikaheka* venom (**A**) into 3FTx- (**B**) and PLA_2_-enriched (**C**) fractions. *In vivo* neutralization assays showed that PLA_2_ molecules represent the main myotoxic components of *M. ikaheka* venom. The estimated LD_50_ for mice of the reverse-phase-isolated 3FTx- (0.22 mg/kg) and PLA_2_- (1.62 mg/kg) enriched fractions, indicated that these two toxin classes contribute synergistically to venom lethality (0.62 mg/kg), with the 3FTxs playing a dominant role [62]. Reproduced with permission from reference [62]. Copyright 2014 Elsevier.

Research on venoms has been continuously enhanced by advances in technology. Progress in high-throughput “omics” methodologies and associated instrumentation has catalyzed an expansion of the scope of biological research from the biochemical analyses of just a few molecules at a time to the systematic and simultaneous study of whole genome sequences, transcriptomes and proteomes. Particularly, the last decade has witnessed the introduction of protocols and strategies for unravelling the toxin composition of venoms (“venomics”) in great detail, both directly (employing proteomics-centered approaches) [20,21,55,56,57] or indirectly (via cDNA library construction or next-generation high-throughput venom gland transcriptomics and bioinformatic analysis) [58,59] in a relatively rapid and cost-effective manner (reviewed in [23]). Proteomics-centered venomics requires homologous searchable databases to fully exploit its analytical capabilities. In addition, the combination of homologous venom proteomics and venom gland transcriptomics can yield complete, or nearly complete, locus-specific, assigned venom proteomes [60,61,62]. Such proteomics characterization of snake venoms offers a framework for rationalizing the bioactivities of venoms (Figure 2) and for clustering venoms based on within- and between-species shared traits and trends [23].

A specific question that has been addressed in recent years using a venomics approach, that is of relevance for the treatment of snakebites, concerns the occurrence and onset of geographic and ontogenetic shifts in toxin composition within *Bothrops* [63,64,65,66], *Crotalus* [25,67,68,69], *Sistrurus* [70,71], *Lachesis* [72,73] and *Gloydius* [74] genera. These studies highlight the concept that a species should be considered as a group of metapopulations and that within a species range, pedomorphic and ontogenetic venom phenotypes often occur in geographically-differentiated areas [23,75]. This knowledge is of fundamental importance for the selection of species and specimens for the manufacturing of improved therapeutic antivenoms. To aid in antivenom design and to assess the range of the possible clinical applications of current commercial or experimental monospecific and polyspecific antivenoms, the proteomics-centered protocol, dubbed “antivenomics”, was developed [76,77].

**Figure 3:**
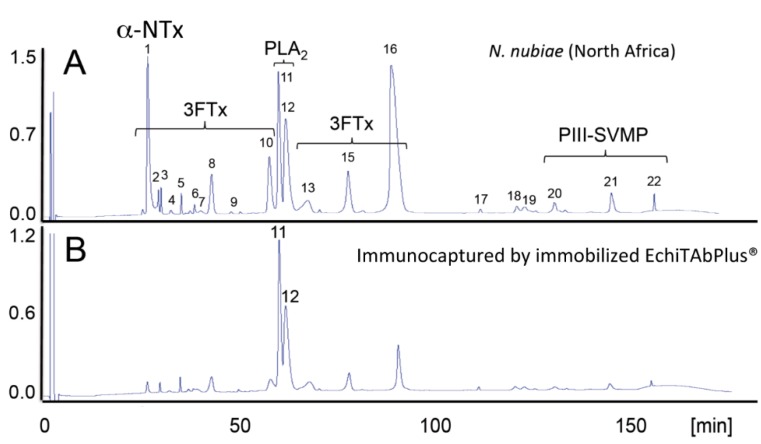

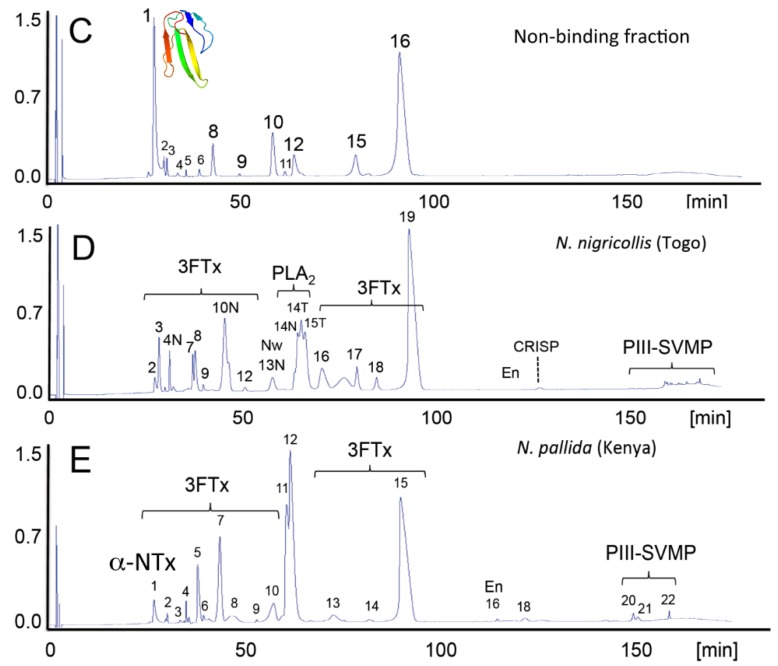
Antivenomics complements neutralization assays. The capability of the Costa Rican antivenom, EchiTAb-Plus-ICP^®^ [81], to reverse the effects of the venoms of African spitting cobras was investigated by neutralization tests [42,77]. EchiTAb-Plus-ICP^®^ neutralized the PLA_2_ and dermonecrotic activities of all of the venoms of African *Naja* snakes sampled. Lethality induced by venoms of the black-necked spitting cobra (*N. nigricollis*), the Mozambique spitting cobra (*N. mossambica*) and the red spitting cobra (*N. pallida*) was eliminated, but did not prevent the lethal effect of the venoms of the Katian spitting cobra (*N. katiensis*) and the Nubian spitting cobra (*N.* *nubiae*). Antivenomics analysis showed that the antivenom immunocaptured PLA_2_ molecules (Peaks 11 and 12 (**B**) and, to a lesser extent, a 3FTx eluted in Peak 16 (**B**), but had impaired the capability of the antivenom to immunodeplete a high abundance type-1 α-neurotoxin (α-NTx) of *N. nubiae* ((**A**) and (**C**); Peak 1, 12.6% of the total venom proteome) and *N. katiensis* (4.4%) venoms correlated with the pre-clinical inability of EchiTAb-Plus-ICP^®^ antivenom to neutralize the lethality of *N.* *nubiae* and *N. katiensis* venoms. Strikingly, although this lethal α-neurotoxin was originally purified “from the venom of *N. nigricollis* collected in Ethiopia in 1961” [82], a recent proteomics survey failed to find α-neurotoxin (6,786.7 Da, SwissProt Accession Code P01426) in *N. nigricollis* venoms [42] (**D**). African spitting cobras have had a long history of taxonomic uncertainty. Relevant for rationalizing the “α-toxin paradox”, *N. nigricollis pallida* was elevated to full species status by Branch [83] and Hughes [84]. This was later supported by Wüster and Broadley [85], who, in addition, described a new species, *N. nubiae*, in populations previously considered to belong to the Katian spitting cobra. α-Neurotoxin is expressed in venoms of *N. pallida* and *N. nubiae* (reverse-phase HPLC Peak 1, in (**A**) and (**E**), respectively), but is virtually absent from the other African spitting *Naja* venoms investigated. Thus, the failure of the EchiTAb-Plus-ICP^®^ antivenom to neutralize the lethal activity of the Nubian and Katian spitting cobra venoms may be due to the absence of α-neurotoxin epitopes in the *N. nigricollis* venom (**D**) employed in the immunization mixture to generate this antivenom. The “α-neurotoxin paradox”, resolved by combining neutralization assays and antivenomics, underpins the importance of getting the taxonomy right for the development of a strategy for the improvement of antivenoms.

Antivenomics complements the *in vitro* and *in vivo* venom activity neutralization assays and traditional immunological methods, such as ELISA and western blot analyses, for assessing the preclinical neutralizing spectrum of antivenoms. Second generation antivenomics [77] is an affinity chromatography protocol to investigate the immuno-capturing ability of immobilized IgG, F(ab')_2_, or Fab antibody molecules followed by the proteomic identification of the venom components recovered in the retained and the non-bound fractions. The antivenomic analysis provides qualitative and also quantitative information on the sets of venom proteins presenting antivenom-recognized epitopes and those exhibiting impaired immunoreactivity. While comparing the levels of immune recognition gathered from antivenomics with the *in vivo* neutralization capacity of an antivenom is not straightforward, since both experiments involve radically different protocols, in our experience, even a moderate immunocapturing capability of ~20%–25% correlates with a good outcome in the *in vivo* neutralization tests. Assuming that the degree of immunorecognition of a toxin by the antivenom’s antibodies represents a measure of the capability of this antivenom to neutralize the toxic activity of that toxin, the antivenomics analysis may assist in assessing the range of clinical applications of current commercial or experimental antivenoms and in the development of improved antivenoms on an immunologically-sound basis. Antivenomics provides thus the grounds for rationalizing the paraspecificity of antivenoms and its capability to aid in the formulation of hypotheses as to how venom mixtures might be designed or re-designed for the manufacturing of improved therapeutic antivenoms. Despite its recent introduction, the usefulness and validity of antivenomics to complement the *in vivo* standard preclinical assays of neutralization of lethality and toxic activities by antivenoms has been documented in a number of investigations in recent years [22,23,42,62,73,78,79,80]. Figure 3 illustrates a practical example of this point.

## 4. Concluding Remarks

The shortage of antivenoms in various parts of the planet can be in part counteracted through the improved deployment of currently existing antivenoms, but also through the design and production of next-generation polyspecific antivenoms of a wide neutralizing spectrum. Venomics-guided uncovering of evolutionary convergent compositional and immunological trends between homologous and heterologous snake venoms may help in the design of such broad specificity antidotes. For this purpose, the choice of immunization mixture cannot be based on phylogenetic distance, but on a deep understanding of the intraspecific venom variation of the medically-important snakes across the geographical range where the antivenom will be deployed. A deep understanding of the venom toxin profile and the identification of divergent and convergent trends along the evolutionary history of venoms have significant implications for selecting the most appropriate species and specimens for manufacturing antivenoms exhibiting an improved therapeutic scope. Antivenomics represents a knowledge-based approach to help in designing improved venom-based immunogen mixtures and to predict paraspecific neutralization by antivenom preparations to the level of species-specific toxins. Accumulating evidence shows the potential of the combination of antivenomics and neutralization assays for analyzing at the molecular level the preclinical efficacy of antivenoms against homologous and heterologous venoms.
